# Alignment-Free Tool for Assemblies and Reads in Genomic Surveillance of Tuberculosis

**DOI:** 10.3201/eid3211.261074

**Published:** 2026-11

**Authors:** Paula Ruiz-Rodriguez, Mireia Coscollá

**Affiliations:** Institute for Integrative Systems Biology, Spanish National Research Council–University of Valencia, FISABIO Joint Research Unit “Infection and Public Health,” Paterna, Spain

**Keywords:** bacteria, respiratory infections, whole-genome sequencing, tuberculosis and other mycobateria, phylogeny, molecular epidemiology, antimicrobial resistance, public health surveillance

## Abstract

Whole-genome sequencing strengthens tuberculosis surveillance, but incomplete lineage schemes can misclassify isolates. We developed Pathotypr, an alignment-free tool for assemblies and reads, using 2,145 markers derived from 26,813 genomes. Across 88,071 samples, Pathotypr improved concordance, expanded uncommon lineage coverage, and detected resistance markers, supporting molecular epidemiology and public health surveillance.

According to the World Health Organization, tuberculosis caused an estimated 1.23 million deaths in 2024 (https://www.who.int/teams/global-programme-on-tuberculosis-and-lung-health/tb-reports/global-tuberculosis-report-2025). Whole-genome sequencing can strengthen disease control by linking cases, detecting resistance, and distinguishing local transmission from repeated introduction; however, those benefits depend on results that are both rapid and comparable. The *Mycobacterium tuberculosis* complex (MTBC) currently includes 10 human-adapted lineages (L1–L10) and 4 animal-adapted clades (A1–A4), and several uncommon or recently described groups remain sparsely sampled or absent from some typing schemes (*1*–*5*). When a scheme does not represent that diversity, isolates may remain unclassified or be assigned to the nearest available label. Differences in sequencing access and reporting remain broader challenges across programs in Europe, but a shared and current lineage framework is still essential wherever genomic surveillance is used (*6*,*7*).

We developed Pathotypr as an open-source, alignment-free software that accepts assembled genomes or raw short reads and returns lineage assignments, World Health Organization–cataloged resistance-associated variants, and a closely related reference genome for downstream analysis. Assemblies can be classified from phylogenetic markers or an independent *k*-mer model, whereas reads are screened directly for lineage and resistance markers and can return >1 supported lineage when mixed signals are present. We evaluated those functions in independent datasets, an 88,071-sample benchmark, and 7,148 genomes from the Comprehensive Resistance Prediction for Tuberculosis: an International Consortium (CRyPTIC) dataset (Appendix).

## The Study

To extract lineage phylomarkers, we obtained a maximum-likelihood phylogeny of 26,813 MTBC genomes and 609,003 polymorphic sites, which placed L1–L10 and A1–A4 within 1 evolutionary backbone and exposed the marked underrepresentation of certain lineages (Figure 1, panel A). We then mapped substitutions to branches and selected single-nucleotide polymorphisms (SNPs) on the stem branch leading to each monophyletic lineage or clade. After removing 3,039 missense variants and 454 homoplastic nonmissense variants, 2,145 of 5,638 candidates remained as lineage-defining markers. A variable overlap of lineage markers occurred with other tools, ranging from no overlap to 698 (62.8%) with TB-Profiler (https://tbdr.lshtm.ac.uk) and 1,750 (12.9%) with SNP-IT (https://github.com/samlipworth/snpit) (Appendix Table 1). Pathotypr lineage-defining markers comprised 33%–46% of total SNPs across lineages, supporting the filtering approach (Figure 1, panel B). However, the small number of genomes for L7–L10 and A1–A3 might be missing diversity not sampled to date.

**Figure 1:**
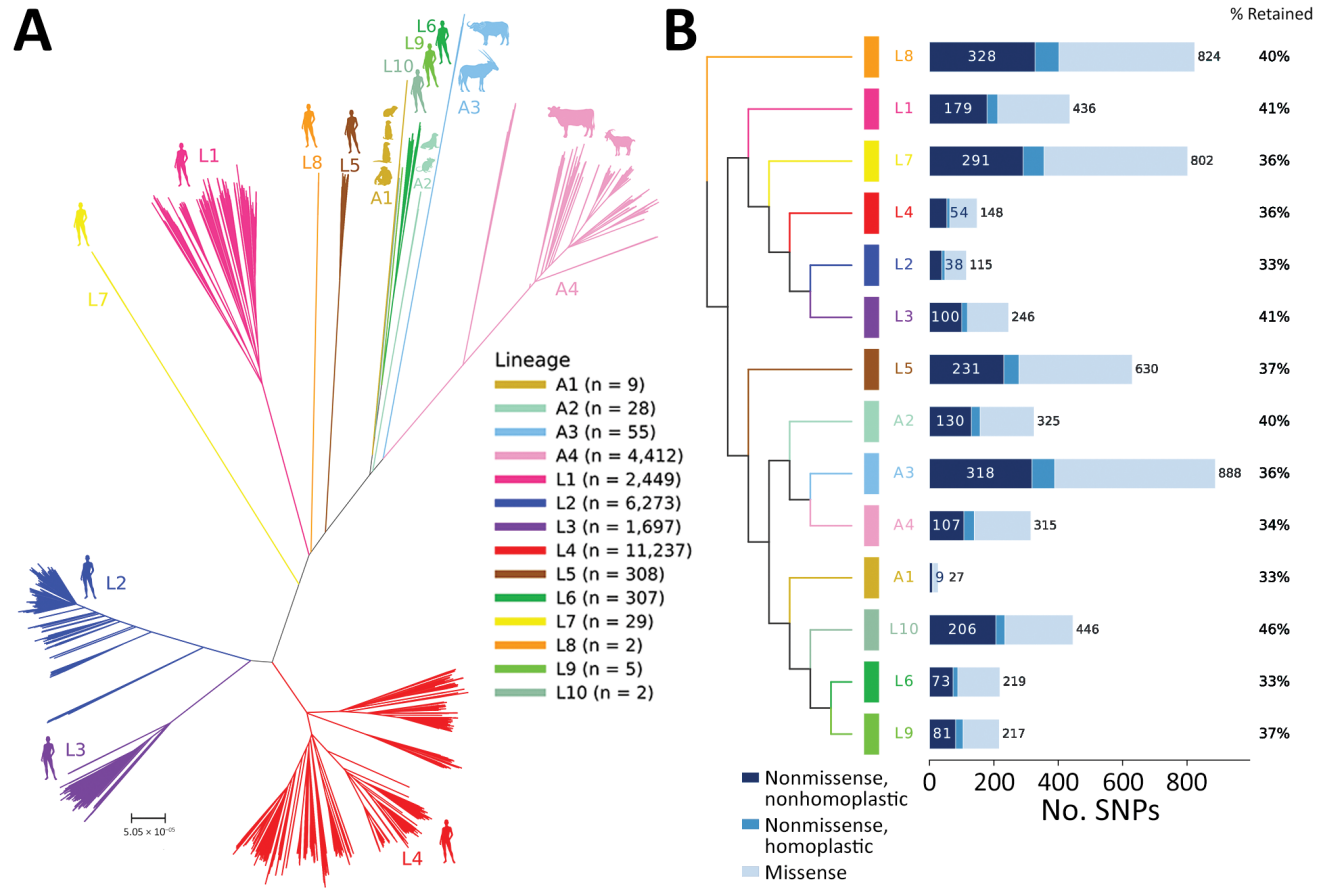
Development and implementation of the lineage framework for Pathotypr, an alignment-free tool for assemblies and reads in genomic surveillance of tuberculosis. A) Maximum-likelihood phylogeny of 26,813 *Mycobacterium tuberculosis* complex genomes, showing L1–L10 and A1–A4. B) Selection of 2,145 lineage-defining markers from 5,638 candidate variants after excluding missense and homoplastic variants. SNP, single-nucleotide polymorphism.

Pathotypr uses 3 modules for lineage assignment (Figure 2). The Predict module assigns lineages to genome assemblies using the *k*-mer–based Random Forest algorithm generated by the Train model, and the Classify and Split-FASTQ modules assign lineages on the basis of SNP markers to assemblies (Classify) and raw sequencing reads (Split-FASTQ). First, we compared lineage assignments by Predict and Classify with the phylogenetic placement of 498 complete RefSeq genomes: Predict matched the phylogenetic lineage in all 498 genomes, and Classify assigned 497. The unassigned genome did not contain the complete marker path required for a call, which could reflect assembly gaps or errors at diagnostic marker positions. Next, Predict and Classify successfully returned the same major-lineage assignment for all 254 chromosome-level assemblies that were not used to train the Random Forest. Finally, Split-FASTQ assigned all 162 clinical read datasets to the expected lineage. Those results show the specific role of each module: Classify provides traceable SNP-based assignments, Predict offers an alternative when the marker path is incomplete, and Split-FASTQ extends the lineage framework directly to short reads (Appendix Table 2, Figure 1).

**Figure 2:**
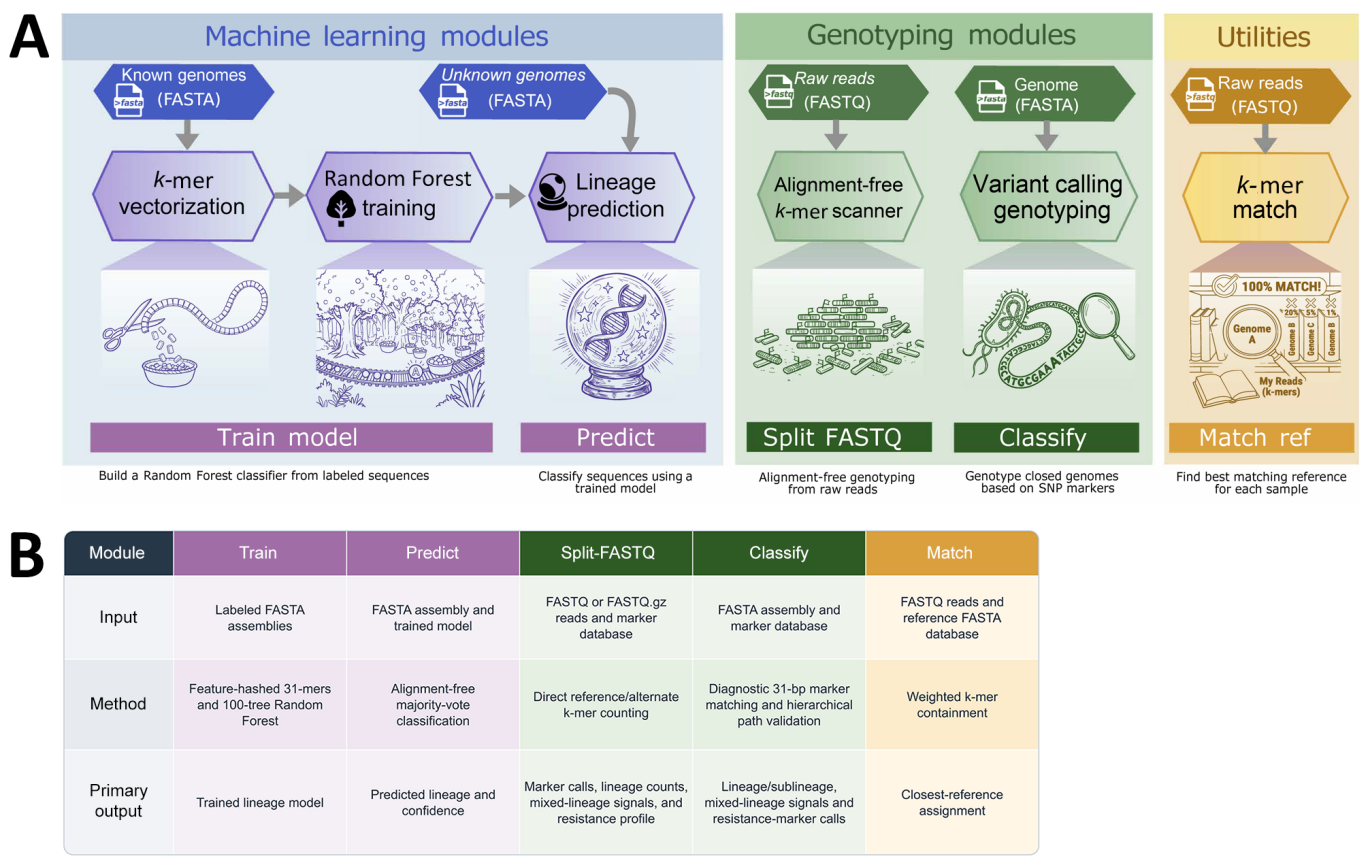
Schematic overview of the 5 modules grouped into 3 functional categories—machine learning, genotyping, and utilities—in developing Pathotypr, an alignment-free tool for assemblies and reads in genomic surveillance of tuberculosis. A) Workflow and data flow across the 5 modules. The Train and Predict modules perform machine learning–based lineage classification; Split-FASTQ and Classify provide alignment-free genotyping from sequencing reads or genome assemblies; and Match identifies the closest reference genome using *k*-mer containment. B) Summary of the input data, analytical method, and primary output of each module. SNP, single-nucleotide polymorphism.

Because the reference choice for read mapping can affect genome coverage and variant calls, we developed and tested Pathotypr’s fourth module, Match Ref, a lineage-aware reference selection (*8*,*9*). Across 162 clinical read sets, mapping to the reference selected by Pathotypr reduced nonfixed SNPs by a median of 89% relative to the MTBC ancestral reference and increased median coverage breadth by 0.84 percentage points (*9*) (Appendix Figure 2).

To test whether the new lineage framework could be applied to surveillance-scale datasets, we typed 88,071 UShER-TB samples (*10*; L.M. Karim et al., unpub. data, https://www.medrxiv.org/content/10.1101/2025.07.22.25331806v1) with Pathotypr module Split-FASTQ (≈24 hours using 4 threads). To compare typing results with TB-Profiler, we excluded 1,290 genomes with multiple lineage calls from the comparison. Although only 698 (62.8%) of the TB-Profiler lineage markers were shared with Pathotypr, both tools assigned the same major lineage to every directly comparable sample (Appendix Table 3). Pathotypr additionally identified 2 L10, 6 A1, and 23 A2 isolates that were not identified with the TB-Profiler marker set. TB-Profiler misclassified 1 L10 isolate as La1.5/*M. bovis* and left the other L10 genome and A1 and A2 genomes unassigned. Compared with 7 other MTBC genomic analysis tools, Pathotypr uniquely combined rapid *k*-mer/Random Forest classification (≈1 s), support for both FASTA and FASTQ file type inputs, drug-resistance prediction, desktop and command-line interfaces, and the broadest lineage coverage (L1–L10 and A1–A4), and still maintained sublineage resolution (Table).

**Table:** Comparison of *Mycobacterium tuberculosis* complex genomic analysis tools, showing main analytical approach, accepted input formats, use of alignment, lineage resolution and taxonomic coverage, drug-resistance reporting, and approximate runtime*

Tool	Reference	Method	Input	AF	Coverage	DR	Runtime†	Interface
Pathotypr	This study	k-mer + RF	FA; FQ(.gz)	Yes	L1–L10; A1–A4	Yes	≈1 s	Desktop; CLI
TB-Profiler	(*10*)	Mapping + SNP DB	FQ; BAM; VCF	No	L1–L9; A3–A4	Yes	3–10 min	Web; CLI
Mykrobe	(*11*)	de Bruijn graph	FQ	Yes	L1–L6	Yes	≈3 min	Desktop; CLI
SNP-IT	(*12*)	SNP panel	FA; VCF	No	L1–L7; animal MTBC	No	1–2 min	CLI
fastlin	(*13*)	Split k-mer	FA; FQ; BAM	Yes	L1–L9; A4	No	<5 s	CLI; binary
KvarQ	(*14*)	Targeted k-mer	FQ	Yes	L1–L7; A4	Yes	≈2 min	Desktop; CLI
MTBseq	(*15*)	Mapping + SNP panel	FQ.gz	No	Major MTBC groups	Yes	≈42 min	CLI
MAGMA	(*16*)	Mapping + cohort calling	PE-FQ	No	TB-Profiler DB	Yes	79/4 h‡	Nextflow; CLI

*AF, alignment-free; CLI, command-line interface; DR, drug-resistance prediction; FA, FASTA; FQ, FASTQ; PE, paired-end; RF, Random Forest; SNP DB, curated SNP database. †Runtime values were obtained with different datasets, hardware, sequencing depths, and software settings and are shown only for context; they do not constitute a controlled benchmark. Only the Pathotypr runtime was measured in this study. ‡Reported for 79 samples processed in ≈4 h.

Pathotypr’s Split-FASTQ classify module complements lineage assignment by reporting World Health Organization–cataloged resistance-associated variants alongside the lineage call in assemblies with different coordinates, as illustrated across the 498-genome validation phylogeny (Appendix Figure 1). For 7,148 CRyPTIC isolates, we generated categorical resistance profiles from World Health Organization confidence groups 1 and 2 and compared them with phenotypic drug-susceptibility results. Genotypic and phenotypic resistance profiles agreed for 86.4% of isolates, and severe disagreement, defined as a difference of >3 levels between the genotypic and phenotypic profiles, occurred in 1.4%. Multidrug-resistant or pre–extensively drug-resistant status was assigned with 93.5% sensitivity and 92.3% positive predictive value. Agreement was lowest for bedaquiline, clofazimine, delamanid, and linezolid, for which most phenotypically resistant isolates carried variants of uncertain significance or no cataloged variant (Figure 3, panel A).

**Figure 3:**
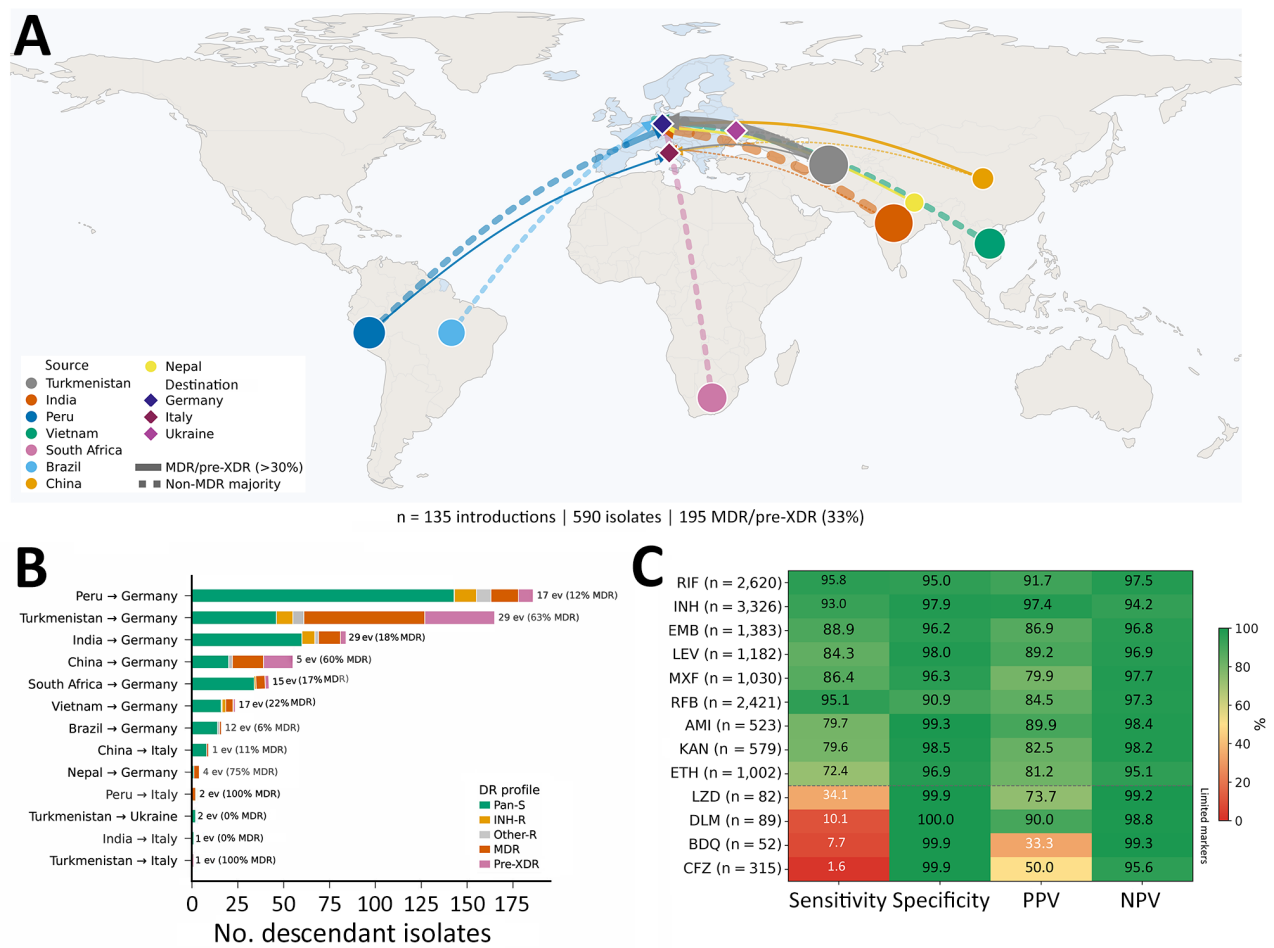
Resistance-informed surveillance analysis of 7,148 CRyPTIC isolates in developing Pathotypr, an alignment-free tool for assemblies and reads in genomic surveillance. A) Probable introductions from non-Europe ancestral states into Germany, Italy, and Ukraine. B) Number and resistance profiles of descendant isolates by source–destination pair. A C) Performance of WHO confidence group 1–2 resistance markers compared with phenotypic drug-susceptibility results. MI, amikacin; BDQ, bedaquiline; CFZ, clofazimine; DLM, delamanid; DR, drug resistance; EMB, ethambutol; ETH, ethionamide; ev, introduction events; INH, isoniazid; KAN, kanamycin; LEV, levofloxacin; LZD, linezolid; MDR, multidrug-resistant; MXF, moxifloxacin; NPV, negative predictive value; pan-S, pansusceptible; PPV, positive predictive value; R, resistant; RFB, rifabutin (genotypic resistance predicted from rifampin-associated variants); RIF, rifampin; XDR, extensively drug-resistant.

We next used the 7,148-isolate CRyPTIC dataset to examine how Pathotypr–Split-FASTQ outputs could contribute to public health surveillance. Lineage assignments formed monophyletic groups in the annotated phylogeny (Appendix Figure 3), and ancestral-state reconstruction inferred 135 probable transitions from non-Europe ancestors into Germany, Italy, or Ukraine, comprising 590 descendant isolates (Figure 3, panels B and C). Among these 590 isolates, 195 (33.1%) had multidrug-resistant or pre–extensively drug-resistant genotypes, compared with 5.5% of the remaining Europe isolates in the dataset. Because CRyPTIC is enriched for drug-resistant isolates, is not population-based, and, after restriction to isolates with a documented country of origin and collection date, includes only Germany, Italy, and Ukraine as recipient countries in Europe, those proportions should not be interpreted as national estimates of importation or transmission. Rather, the analysis illustrates how standardized lineage and resistance outputs can be combined with other tools to identify cross-border clusters that merit epidemiologic follow-up (*17*).

## Conclusions

The central scientific advance of this study is a phylogeny-derived lineage framework that represents the recognized diversity of the MTBC, with Pathotypr as the software system we developed to make that framework usable in genomic surveillance. This system’s 2,145 markers provide a traceable and extensible basis for assigning common and uncommon, human-adapted and animal-adapted groups, and the independent *k*-mer model preserves classification when direct marker detection is incomplete. By analyzing assemblies and reads without a conventional mapping workflow, reporting multiple supported lineages in mixed samples, and selecting a closely related reference genome, Pathotypr reduces unresolved or inappropriate assignments and places resistance results within a consistent evolutionary context.

Broad lineage coverage, rapid processing, standardized resistance reporting, and lineage-aware reference selection make Pathotypr a practical route from sequence data to comparable surveillance results. Our platform’s low memory requirements, desktop graphical interface, and command-line implementation make the same workflow accessible to laboratories with limited bioinformatics infrastructure while supporting automated analysis of large collections. Those features can improve reporting across laboratories, recognition of uncommon introductions, outbreak review, and prioritization of resistant cross-border clusters.

Appendix Additional information for alignment-free tool for assemblies and reads in genomic surveillance of tuberculosis.
