# Supplementary material for: Alignment-Free Tool for Assemblies and Reads in Genomic Surveillance of Tuberculosis
Source: Emerg Infect Dis. 2026 Nov;32(11):1913–8. doi: 10.3201/eid3211.261074 (PMC13715872; doi:10.3201/eid3211.261074)
Supplement: Supplementary file 1 [file 26-1074-Techapp-s1.pdf]

Article DOI: http://doi.org/10.3201/eid3211.261074

EID cannot ensure accessibility for supplementary materials supplied by authors. Readers who have difficulty accessing supplementary content should contact the authors for assistance.

Alignment-Free Tool for Assemblies and Reads in Genomic Surveillance of Tuberculosis

Appendix

Study Design and Datasets

We developed Pathotypr as an alignment-free framework for *Mycobacterium tuberculosis* complex (MTBC) lineage assignment, resistance-associated variant detection, and closest-reference selection from assemblies and raw reads. The study combined phylogenetic reconstruction and marker derivation from 26,813 genomes with validation in 498 complete RefSeq assemblies, 254 independent chromosome-level assemblies, and 162 clinical read datasets. We also evaluated closest-reference selection against a panel of 500 complete assemblies, benchmarked lineage assignment in 88,071 UShER-TB samples, and analyzed resistance and phylogeographic patterns in 7,148 CRyPTIC isolates. Appendix Table 4 summarizes the datasets; accession-level results are provided in the deposited supplementary files Appendix Table 5.

Phylogenetic Reconstruction and Lineage Markers

We extracted polymorphic sites relative to the inferred MTB_ancestor reference with SNPick version 0.1.0 (https://github.com/PathoGenOmics-Lab/snpick), excluding previously reported problematic sites and positions associated with drug resistance (*1*). The final alignment contained 609,003 polymorphic sites from 26,813 genomes.

We reconstructed a maximum-likelihood phylogeny with IQ-TREE version 2.0.0 under a GTR+G4 model with ascertainment-bias correction and inferred branch-specific substitutions with TreeTime version 0.11.4 (*2*,*3*). Candidate markers were SNPs mapped to the stem branch

leading to each monophyletic lineage or animal-adapted clade. We removed homoplastic variants and annotated the remaining sites against H37Rv with SnpEff version 5.1 (*4*). After excluding 3,039 missense variants and 454 homoplastic non-missense variants, 2,145 of 5,638 candidates remained as lineage-defining markers for L1–L10 and A1–A4. Marker retention ranged from 33% to 46% across groups, indicating no marked lineage-specific loss during filtering, although L7–L10 and A1–A3 remained sparsely sampled.

The 2,145 markers are the newly derived major-lineage and clade markers reported in this study. Pathotypr also contains previously published diagnostic SNPs for finer sublineage assignment; those established markers are not presented as a new contribution.

Pathotypr Implementation

Pathotypr was written in Rust and is distributed as a command-line program and a cross-platform desktop application. Both interfaces use the same analytical core and accept FASTA assemblies and FASTQ or compressed FASTQ reads. Appendix Table 2 summarizes the five components and their outputs.

Train represents labeled assemblies as feature-hashed 31-mer vectors and fits a 100-tree Random Forest using an 80:20 training:test split and random seed 42. Predict applies the trained model to an assembly and reports the majority-vote lineage and model support.

Classify searches assemblies for diagnostic 31-bp sequences surrounding lineage and resistance markers. It reports the deepest lineage or sublineage supported by a complete hierarchical marker path; assemblies without a valid path remain unclassified rather than being forced into the nearest label.

Split-FASTQ counts reference and alternate diagnostic k-mers directly in reads. Marker-level results include position, allele counts, allele fraction, and associated lineage. When qualifying markers support more than one lineage, Pathotypr retains each supported lineage and flags the sample as containing mixed-lineage signals. It does not estimate strain proportions or reconstruct the component genomes.

Match compares read k-mers with a reference database and returns the assembly with the highest weighted containment score. It recommends a reference for later mapping but does not itself perform alignment or variant calling.

Read-Calling Thresholds

Split-FASTQ requires a minimum combined depth of 10 reads and an alternate-allele fraction of at least 95%. The thresholds operate jointly as conservative criteria for nearly fixed lineage and resistance markers rather than as a biologic detection limit.

At depths of 10–19 reads, the 95% criterion requires every informative read to support the alternate allele; at 20×, at least 19 of 20 reads must support it. A 90% threshold was considered during development, but we increased it to 95% to reduce borderline fixed-marker calls caused by sequencing error, mapping noise, or low-level heterogeneity. A 10× minimum also has methodological precedent in the Snippy workflow used by SNP-IT, which combined 10× depth with 90% allele frequency (*5*).

The 10× cutoff was not optimized in a dedicated downsampling analysis. We therefore describe it as a conservative operational threshold whose adequacy was supported by its consistent application in the clinical-read validation, in which all 162 datasets were assigned to the expected lineage.

Resistance Interpretation

Pathotypr screened resistance-associated variants represented in the WHO mutation catalog used to construct the versioned resistance-marker file (*6*). The WHO catalog assigns confidence groups to mutations rather than assigning tiers to genes. Pathotypr screened all genes and genomic regions represented in the supplied marker file, but only confidence groups 1 and 2 determined categorical resistance profiles.

Group 3 variants have uncertain significance, whereas groups 4 and 5 are not considered evidence of resistance. Variants in groups 3–5 are reported in the output by Pathotypr but not considered in the current analysis. Uncatalogued variants were not interpreted as established

resistance determinants, and Pathotypr did not apply additional correlation with phenotype-based, or epistatic grading rules.

For the CRyPTIC comparison, genotypic and phenotypic results were grouped into five ordered categories: pansusceptible, isoniazid-resistant, other-resistant, multidrug-resistant, and pre-extensively drug-resistant. The absolute distance between the genotypic and phenotypic categories was used to classify agreement as concordant, minor, moderate, or severe.

Validation and Large-Scale Benchmarking

We compared Predict and Classify assignments with maximum-likelihood phylogenetic placement in 498 complete RefSeq genomes. Predict agreed with phylogenetic placement for all 498 genomes, whereas Classify agreed for 497 and left one genome unclassified because it did not contain the complete marker path required for a call. Predict assigned that genome to the lineage indicated by the phylogeny.

Predict and Classify produced the same major-lineage assignment for all 254 chromosome-level assemblies withheld from model development. Split-FASTQ assigned all 162 clinical read datasets to the expected lineage. Appendix Table 2 summarizes these evaluations, and Appendix Figure 1 shows the complete validation phylogeny.

TB-Profiler was selected for the 88,071-sample benchmark because UShER-TB provided precomputed sample-level calls at the required scale and because TB-Profiler reports both lineage and resistance information (*7*) (Karim LM et al, unpub. data, https://www.medrxiv.org/content/10.1101/2025.07.22.25331806v1.abstract). The most important features of a typing scheme were compared across eight MTBC lineage-typing tools in Table 1, including broader mapping and phylogenomic pipelines (*8*,*9*).

Closest-Reference Evaluation

We evaluated Match in 162 paired-end clinical read datasets against a panel of 500 complete RefSeq assemblies. Reads were mapped to both the Pathotypr-selected reference and the inferred ancestral MTBC reference (https://zenodo.org/records/3497110) with bwa-mem2 version 2.3, and variants were called with bcftools version 1.22 (*10*). We compared lineage–

reference concordance, mapped reads, coverage breadth and depth, error rate, and fixed and nonfixed SNP counts. For this downstream mapping analysis, fixed SNPs had an alternate-allele fraction ≥0.90 and nonfixed SNPs an allele fraction of 0.05–0.90; these definitions are separate from the 95% Pathotypr marker threshold.

CRyPTIC Analysis

Isolates were selected from the CRyPTIC isolates (*11*) based only on metadata completeness and sequencing quality, with no geographic criterion. We required a documented country of origin for migration reconstruction and a day-resolved collection date for time-scaled phylogeny, excluding records dated 1 January when this indicated year-only resolution. Of 12,289 isolates, 9,005 had both fields; 7,840 paired-end datasets were retrieved, 7,148 passed quality control (mean depth ≥30×, ≥90% reads mapped, ≥100,000 reads after trimming, ≤500 heterozygous variants and ≤20% duplication).

Pathotypr assigned lineages and resistance using WHO confidence groups 1 and 2, and drug-specific sensitivity, specificity, positive predictive value and negative predictive value were calculated against phenotypic susceptibility testing.

We reconstructed a maximum-likelihood phylogeny with IQ-TREE under a GTR+G4 model and inferred ancestral geographic states with TreeTime migration. Probable introductions were defined as transitions from a reconstructed non-European ancestral state into Germany, Italy, or Ukraine. Descendant isolates were assigned to the corresponding event, and enrichment of multidrug-resistant or pre-extensively drug-resistant genotypes was evaluated with a 2-sided Fisher exact test.

CRyPTIC is enriched for drug-resistant isolates, is not population-based, and includes only Germany, Italy, and Ukraine as European recipient countries. The analysis therefore illustrates a surveillance application and does not estimate national importation or transmission burdens.

Comparison with Published Lineage-Marker Schemes

To characterize how the Pathotypr marker database relates to existing lineage schemes, we compared its 3,706 distinct positions, with 11 published MTBC SNP panels (Appendix Table 1). Marker lists were obtained from the original publications, supplementary files, or public software repositories. We calculated overlap at the level of distinct genomic positions and expressed it as a percentage of each comparator panel. Because Pathotypr markers are polarized to the inferred ancestor, whereas most comparator panels are polarized to H37Rv, allele agreement was evaluated using the derived allele. Positional overlap was interpreted as a measure of marker-selection strategy and taxonomic scope, rather than as a measure of classification accuracy, which was assessed separately in the sample-level benchmark.

Pathotypr can assign sublineages using previously published diagnostic SNPs included in its marker database. However, those sublineage markers were not newly derived in this study, and the formal benchmark was focused on the new L1–L10 and A1–A4 framework. Differences in sublineage names, marker hierarchies, and nesting rules also prevent a simple label-by-label comparison from being interpreted as biologic agreement. For the same reason, raw SNP overlap was not treated as proof of functional equivalence: different schemes can reach compatible classifications using different diagnostic positions and hierarchical rules. The complete Pathotypr marker file is publicly available in https://zenodo.org/records/19210043 for independent comparison.

References

1. Modlin SJ, Robinhold C, Morrissey C, Mitchell SN, Ramirez-Busby SM, Shmaya T, et al. Exact mapping of Illumina blind spots in the *Mycobacterium tuberculosis* genome reveals platform-wide and workflow-specific biases. Microb Genom. 2021;7:mgen000465. PubMed https://doi.org/10.1099/mgen.0.000465

2. Nguyen LT, Schmidt HA, von Haeseler A, Minh BQ. IQ-TREE: a fast and effective stochastic algorithm for estimating maximum-likelihood phylogenies. Mol Biol Evol. 2015;32:268–74. PubMed https://doi.org/10.1093/molbev/msu300

3. Sagulenko P, Puller V, Neher RA. TreeTime: Maximum-likelihood phylodynamic analysis. [cited 2025 Jan 26]. Virus Evol. 2018;4:vex042. PubMed https://doi.org/10.1093/ve/vex042

4. Cingolani P, Platts A, Wang L, Coon M, Nguyen T, Wang L, et al. A program for annotating and predicting the effects of single nucleotide polymorphisms, SnpEff. [cited 2024 Oct 26]. Fly (Austin). 2012;6:80–92. PubMed https://doi.org/10.4161/fly.19695

5. Lipworth S, Jajou R, de Neeling A, Bradley P, van der Hoek W, Maphalala G, et al. SNP-IT tool for identifying subspecies and associated lineages of *Mycobacterium tuberculosis* complex. Emerg Infect Dis. 2019;25:482–8. PubMed https://doi.org/10.3201/eid2503.180894

6. World Health Organization. Catalogue of mutations in *Mycobacterium tuberculosis* complex and their association with drug resistance. World Health Organization; 2023 [cited 2026 Mar 23]. https://www.who.int/publications/i/item/9789240082410

7. Phelan JE, O'Sullivan DM, Machado D, Ramos J, Oppong YEA, Campino S, et al. Integrating informatics tools and portable sequencing technology for rapid detection of resistance to anti-tuberculous drugs. Genome Med. 2019;11:41. PubMed https://doi.org/10.1186/s13073-019-0650-x

8. Kohl TA, Utpatel C, Schleusener V, De Filippo MR, Beckert P, Cirillo DM, et al. MTBseq: a comprehensive pipeline for whole genome sequence analysis of *Mycobacterium tuberculosis* complex isolates. [cited 2026 July 20]. PeerJ. 2018;6(e5895):e5895. PubMed https://doi.org/10.7717/peerj.5895

9. Heupink TH, Verboven L, Sharma A, Rennie V, de Diego Fuertes M, Warren RM, et al. The MAGMA pipeline for comprehensive genomic analyses of clinical Mycobacterium tuberculosis samples. PLOS Comput Biol. 2023;19:e1011648. PubMed https://doi.org/10.1371/journal.pcbi.1011648

10. Danecek P, Bonfield JK, Liddle J, Marshall J, Ohan V, Pollard MO, et al. Twelve years of SAMtools and BCFtools. Gigascience. 2021;10:giab008. PubMed https://doi.org/10.1093/gigascience/giab008

11. The CRyPTIC Consortium. A data compendium associating the genomes of 12,289 *Mycobacterium tuberculosis* isolates with quantitative resistance phenotypes to 13 antibiotics. PLoS Biol. 2022;20:e3001721. PubMed https://doi.org/10.1371/journal.pbio.3001721

12. Derelle R, Lees J, Phelan J, Lalvani A, Arinaminpathy N, Chindelevitch L. fastlin: an ultra-fast program for *Mycobacterium tuberculosis* complex lineage typing. Bioinformatics. 2023;39:btad648. PubMed https://doi.org/10.1093/bioinformatics/btad648

13. Zwyer M, Çavusoglu C, Ghielmetti G, Pacciarini ML, Scaltriti E, Van Soolingen D, et al. A new nomenclature for the livestock-associated *Mycobacterium tuberculosis* complex based on

phylogenomics. Open Res Eur. 2021;1:100. PubMed https://doi.org/10.12688/openreseurope.14029.2

14. Coll F, McNerney R, Guerra-Assunção JA, Glynn JR, Perdigão J, Viveiros M, et al. A robust SNP barcode for typing *Mycobacterium tuberculosis* complex strains. Nat Commun. 2014;5:4812. PubMed https://doi.org/10.1038/ncomms5812

15. Hunt M, Bradley P, Lapierre SG, Heys S, Thomsit M, Hall MB, et al. Antibiotic resistance prediction for *Mycobacterium tuberculosis* from genome sequence data with Mykrobe. [cited 2026 Mar 24]. Wellcome Open Res. 2019;4:191. PubMed https://doi.org/10.12688/wellcomeopenres.15603.1

16. Freschi L, Vargas R Jr, Husain A, Kamal SMM, Skrahina A, Tahseen S, et al. Population structure, biogeography and transmissibility of *Mycobacterium tuberculosis*. Nat Commun. 2021;12:6099. PubMed https://doi.org/10.1038/s41467-021-26248-1

17. Steiner A, Stucki D, Coscolla M, Borrell S, Gagneux S. KvarQ: targeted and direct variant calling from fastq reads of bacterial genomes. [cited 2026 Jan 24]. BMC Genomics. 2014;15:881. PubMed https://doi.org/10.1186/1471-2164-15-881

18. Shitikov E, Kolchenko S, Mokrousov I, Bespyatykh J, Ischenko D, Ilina E, et al. Evolutionary pathway analysis and unified classification of East Asian lineage of *Mycobacterium tuberculosis*. Sci Rep. 2017;7:9227. PubMed https://doi.org/10.1038/s41598-017-10018-5

19. Stucki D, Brites D, Jeljeli L, Coscolla M, Liu Q, Trauner A, et al. *Mycobacterium tuberculosis* lineage 4 comprises globally distributed and geographically restricted sublineages. Nat Genet. 2016;48:1535–43. PubMed https://doi.org/10.1038/ng.3704

Appendix Table 1. Comparison of the Pathotypr lineage-marker panel with published MTBC lineage-signature schemes in developing Pathotypr: alignment-free tool for assemblies and reads in genomic surveillance

Scheme	Reference	Total SNPs, n	Shared with Pathotypr, n (%)*	Main reason for differences
**SNP-IT**	(*5*)	13,602	1,750 (12.9)	Exhaustive branch enumeration; approximately half of the positions classify *M. canettii*
**fastlin**	(*12*)	1,230	713 (58.0)	Approximately 10 markers per node; largely re-encodes the TB-Profiler scheme
**TB-Profiler**	(*7*)	1,111	698 (62.8)	Approximately 10 markers per major lineage; the shared marker set is nested within fastlin
**Zwyer et al.**	(*13*)	1,867	333 (17.8)	Designed specifically for livestock-associated MTBC clades
**MTBseq**	(*8*)	96	24 (25.0)	Small diagnostic set assembled from previously published barcodes
**Coll et al.**	(*14*)	62	21 (33.9)	Minimal barcode, including one SNP each for L5, L6, L7, and *M. bovis*
**Mykrobe**	(*15*)	70	21 (30.0)	Small panel derived from earlier lineage barcodes
**Freschi et al.**	(*16*)	95	18 (18.9)	Restricted to human-adapted lineages
**KvarQ**	(*17*)	27	7 (25.9)	Legacy marker set developed before the description of L8–L10
**Shitikov et al.**	(*18*)	21	5 (23.8)	Restricted to L2/Beijing sublineages
**Stucki et al.**	(*19*)	8	0 (0.0)	Eight-marker panel designed specifically for L4 sublineages

Appendix Table 2. Validation of Pathotypr lineage assignment and closest-reference selection in developing Pathotypr: alignment-free tool for assemblies and reads in genomic surveillance*

Evaluation	No. samples	Result
Predict vs. phylogenetic placement	498	498/498 concordant
Classify versus phylogenetic placement	498	497/498 concordant; 1 unclassified
Predict versus Classify in independent assemblies	254	254/254 concordant
Split-FASTQ versus expected clinical-read lineage	162	162/162 concordant
Match-selected reference versus sample lineage	162	157/162 concordant; all discordant selections were A1
Marker-unclassified genome	1	Incomplete marker path; Predict agreed with phylogenetic placement

*Table summarizes agreement between Pathotypr and phylogenetic placement in complete genomes, agreement between the marker- and model-based approaches in independent assemblies, direct lineage assignment in clinical reads, and lineage concordance of the selected reference genomes.

Appendix Table 3. Major-lineage comparison of Pathotypr and TB-Profiler in 88,071 UShER-TB samples in developing Pathotypr: alignment-free tool for assemblies and reads in genomic surveillance*

Lineage	Pathotypr, n	TB-Profiler, n	Concordance, %	Markers (mean, range)	Pathotypr time	TB-Profiler time
L1	9 012	9 012	100.0	172 (2–204)	2.6 h	64 d
L2	28 263	28 263	100.0	71 (1–94)	8.3 h	208 d
L3	8 967	8 967	100.0	73 (1–110)	2.6 h	64 d
L4	36 118	36 118	100.0	99 (1–162)	10.4 h	261 d
L5	370	370	100.0	238 (3–253)	6 min	3 d
L6	847	847	100.0	89 (7–111)	15 min	6 d
L7	39	39	100.0	273 (158–277)	39 s	6.5 h
L8	2	2	100.0	199 (143–255)	2 s	20 min
L9	24	24	100.0	63 (10–66)	25 s	4.2 h
L10‡	2	0	0	120 (117–122)	2 s	NA
A1‡	6	0	0	143 (1–312)	6 s	NA
A2‡	23	0	0	119 (3–187)	23 s	NA
A3	198	198	100.0	289 (46–311)	4 min	1 d
A4§	4 200	4 200	100.0	272 (24–299)	1.2 h	29 d
**Total**	**88 071**	**88 049**	**99.9**		**~1 d**	**~636 d**

*Table summarizes agreement among single-lineage samples belonging to groups represented in both schemes, the additional L10, A1, and A2 assignments made by Pathotypr. Groups absent from the TB-Profiler marker set were reported separately and were not counted as disagreements. Mean marker counts are shown as mean (range) lineage-defining SNPs per sample used in Pathotypr. Estimated runtimes were ≈1 s per sample for Pathotypr and 10 min per sample for TB-Profiler using four threads.
‡ TB-Profiler lacks lineage 10, A1, A2 markers; one L10 sample was classified as La1.5 (M. bovis) and the other one of L10, all A1 and A2 were unassigned.
§ Includes 51 M. caprae samples (TB-Profiler La2), counted as concordant within A4.
NA, not applicable.

Appendix Table 4. Datasets used to develop, validate, benchmark, and apply Pathotypr in developing Pathotypr: alignment-free tool for assemblies and reads in genomic surveillance*

Dataset	n	Purpose	Pathotypr module(s)
MTBC phylogenetic dataset	26,813	Phylogenetic reconstruction and major-lineage marker derivation	Marker-development workflow
Complete RefSeq assemblies	498	Model development and lineage validation	Predict; Classify
Independent chromosome-level assemblies	254	Generalizability assessment	Predict; Classify
Clinical paired-end read sets	162	Direct-read lineage validation and closest-reference evaluation	Split-FASTQ; Match
Complete RefSeq reference panel	500	Candidate references for read mapping	Match
UShER-TB benchmark	88,071	Large-scale comparison with TB-Profiler	Split-FASTQ
CRyPTIC application	7,148	Resistance validation and phylogeographic surveillance application	Split-FASTQ plus phylogenetic analysis

*Table lists the number of samples in each dataset, its analytical purpose, and the Pathotypr component or downstream analysis applied. The datasets include the phylogenetic collection, complete and independent assemblies, clinical read sets, the closest-reference panel, the UShER-TB benchmark, and the CRyPTIC surveillance application. MTBC, *Mycobacterium tuberculosis* complex.

Appendix Table 5. Supplementary datasets deposited in Zenodo in developing Pathotypr: alignment-free tool for assemblies and reads in genomic surveillance*

Dataset	Contents
Supplementary dataset 1	Metadata for the 26,813 genomes used for phylogenetic reconstruction and marker derivation
Supplementary dataset 2	Pathotypr assignments for complete and independent chromosome-level assemblies, including the marker-unclassified genome
Supplementary dataset 3	Pathotypr and TB-Profiler outputs for the 88,071 UShER-TB samples
Supplementary dataset 4	Closest-reference and ancestral-reference mapping metrics for 162 clinical read datasets
Supplementary dataset 5	CRyPTIC phenotypic and genotypic resistance profiles, metadata, and transition annotations
Supplementary dataset 6	Pathotypr lineage and resistance-marker results for the 162 clinical read datasets
Supplementary dataset 7	Resistance-associated variants detected in the 7,148 CRyPTIC isolates

*Table lists each deposited dataset and summarizes its contents, including genome metadata, validation results, UShER-TB benchmark outputs, closest-reference metrics, CRyPTIC resistance and epidemiologic data, and detected resistance-associated variants. Complete sample-level files are available in Zenodo under https://doi.org/10.5281/zenodo.20630545.

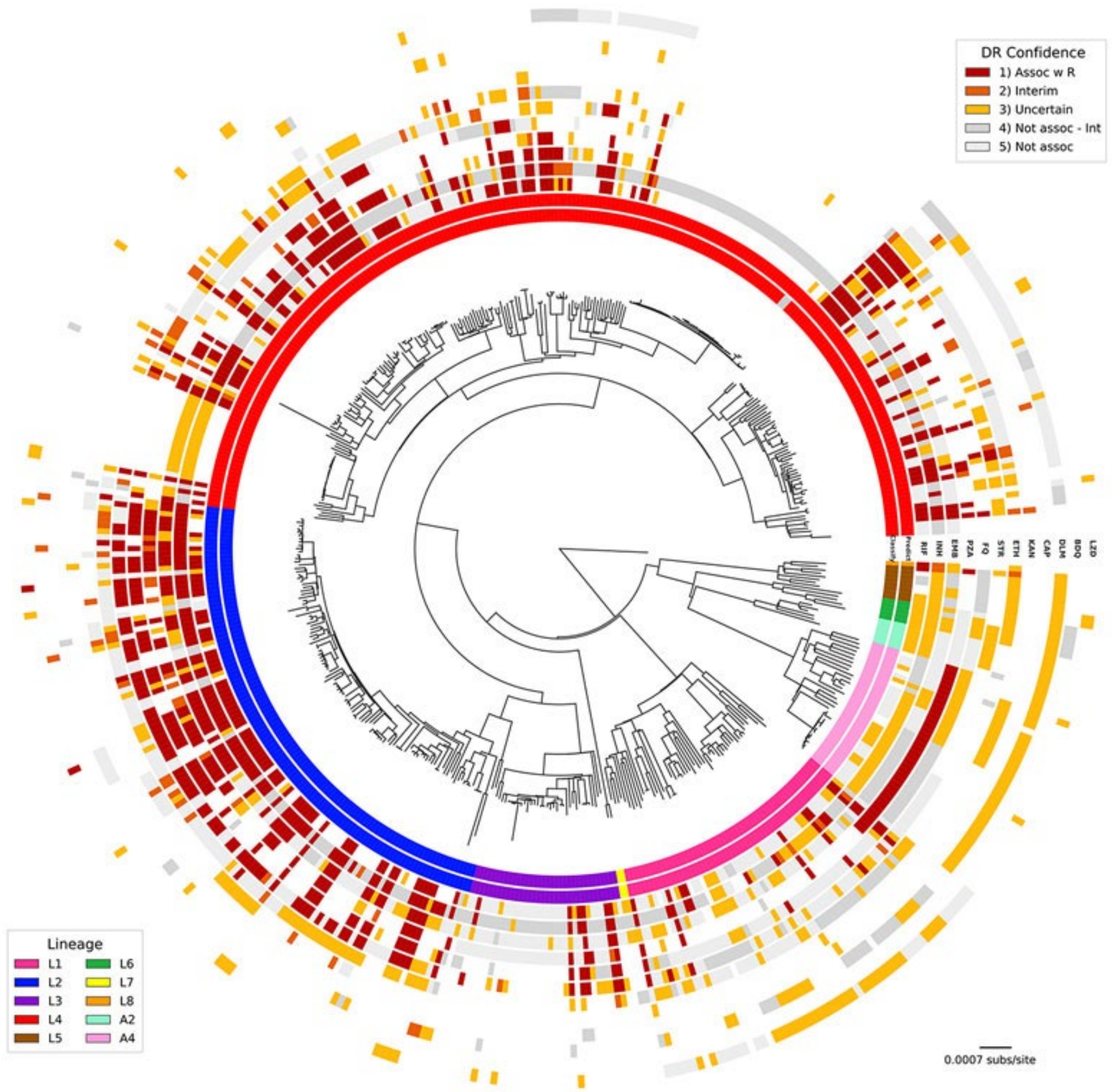

Appendix Figure 1. Phylogenetic validation of Pathotypr in 498 complete RefSeq genomes in developing Pathotypr: alignment-free tool for assemblies and reads in genomic surveillance. Maximum-likelihood phylogeny annotated with lineage assignments from the marker-based Classify and *k*-mer–based Predict approaches and with World Health Organization–cataloged resistance variants by drug and confidence group. Predict agreed with phylogenetic placement for all 498 genomes; Classify agreed for 497 and left 1 unclassified. BDQ, bedaquiline; CAP, capreomycin; DLM, delamanid; EMB, ethambutol; ETH, ethionamide; FQ, fluoroquinolones; INH, isoniazid; KAN, kanamycin; LZD, linezolid; PZA, pyrazinamide; RIF, rifampin; STR, streptomycin.

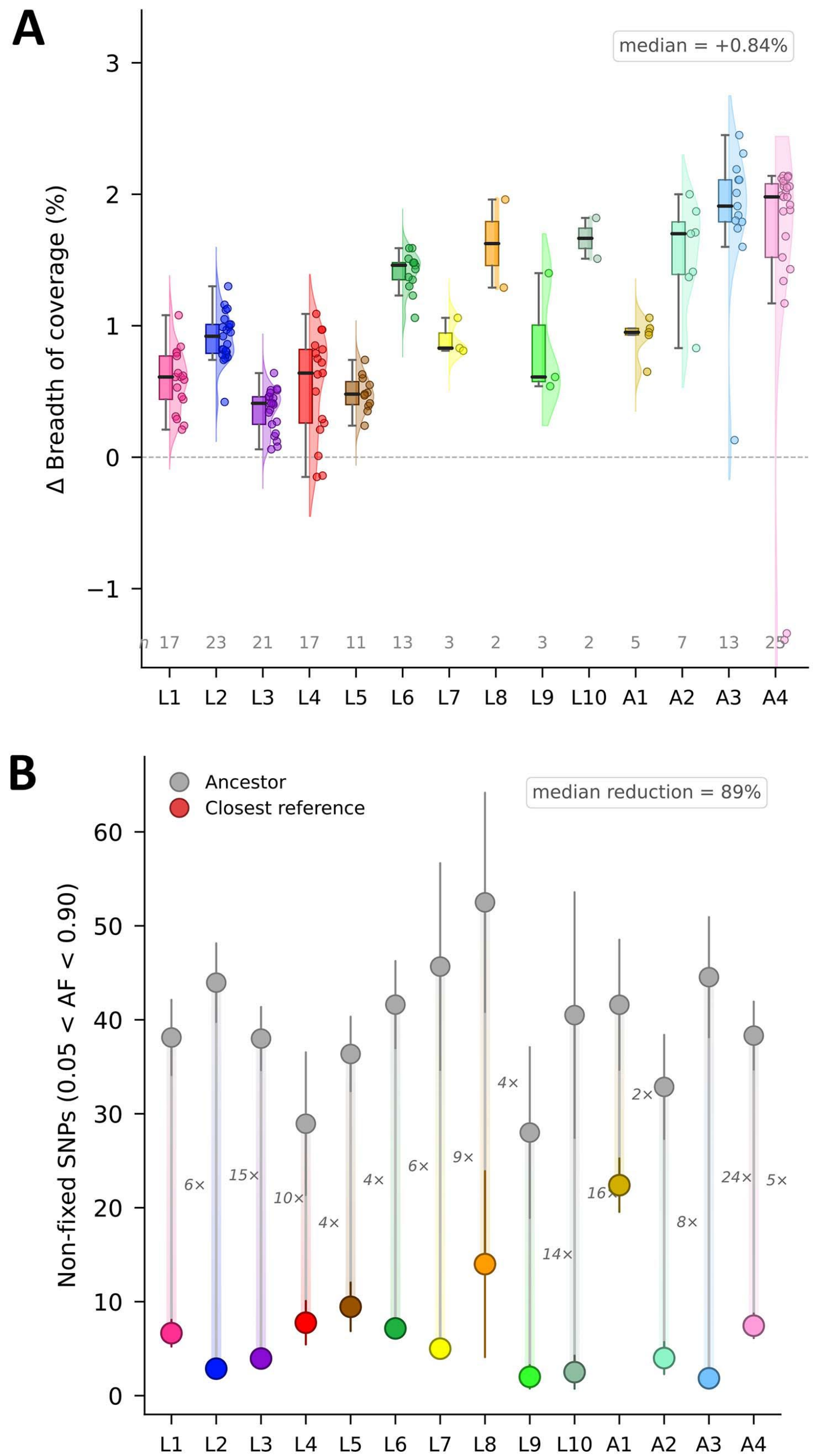

Appendix Figure 2. Effect of lineage-aware reference selection in 162 clinical read datasets in developing Pathotypr: alignment-free tool for assemblies and reads in genomic surveillance. A) Change in genome coverage after mapping reads to the Pathotypr-selected reference rather than the ancestral *Mycobacterium tuberculosis* complex reference; positive values indicate broader coverage with the selected reference. B) Nonfixed single-nucleotide polymorphism counts obtained with both references. Pathotypr-selected references reduced the median number of nonfixed single-nucleotide polymorphisms by 89%.

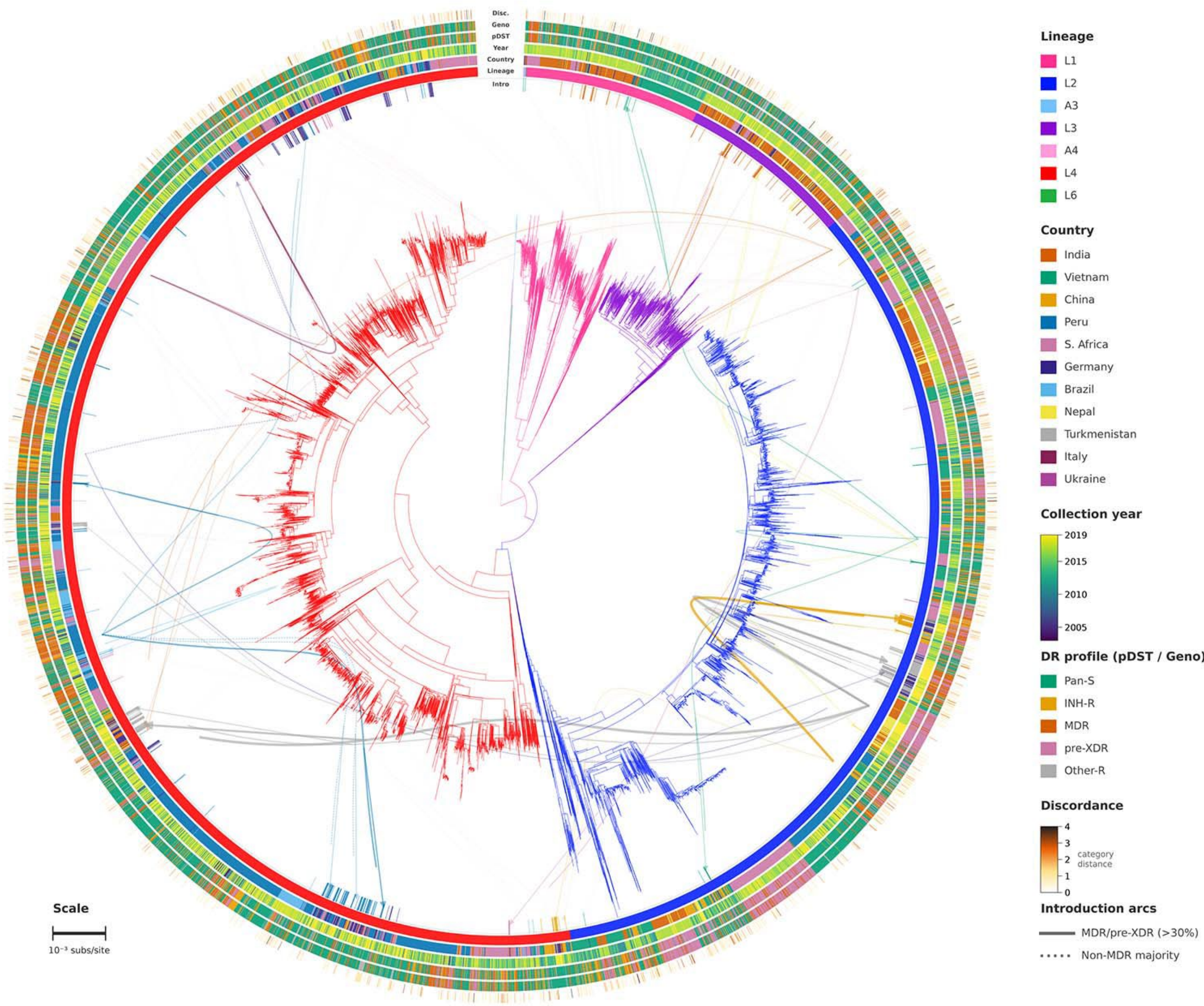

Appendix Figure 3. Phylogenetic distribution of lineage, geographic, temporal, and resistance data among 7,148 CRyPTIC isolates in developing Pathotypr: alignment-free tool for assemblies and reads in genomic surveillance**.** Branches colored by Pathotypr lineage assignment. Annotation rings show country, collection year, phenotypic and genotypic resistance profiles, and genotype–phenotype category distance. Interior arcs mark probable transitions into Germany, Italy, or Ukraine; solid arcs indicate clades with >30% multidrug resistant/pre–extensively drug-resistant genotypes. Dotted arcs indicate a non–multidrug resistant majority.
